# Nutritional Manipulation of One-Carbon Metabolism: Effects on Arsenic Methylation and Toxicity

**DOI:** 10.1155/2012/595307

**Published:** 2012-03-14

**Authors:** Megan N. Hall, Mary V. Gamble

**Affiliations:** ^1^Department of Epidemiology, Mailman School of Public Health, Columbia University, 722 West 168th Street, Room T31, New York, NY 10032, USA; ^2^Department of Environmental Health Sciences, Mailman School of Public Health, Columbia University, 722 West 168th Street, Room 1107E, New York, NY 10032, USA

## Abstract

Exposure to arsenic (As) through drinking water is a substantial problem worldwide. The methylation of As, a reactive metalloid, generates monomethyl- (MMA) and dimethyl-arsenical (DMA) species. The biochemical pathway that catalyzes these reactions, one-carbon metabolism, is regulated by folate and other micronutrients. Arsenic methylation exerts a critical influence on both its urinary elimination and chemical reactivity. Mice having the As methyltransferase null genotype show reduced urinary As excretion, increased As retention, and severe systemic toxicity. The most toxic As metabolite *in vitro* is MMA^III^, an intermediate in the generation of DMA^V^, a much less toxic metabolite. These findings have raised the question of whether As methylation is a detoxification or bioactivation pathway. Results of population-based studies suggest that complete methylation of inorganic As to DMA is associated with reduced risk for As-induced health outcomes, and that nutrients involved in one-carbon metabolism, such as folate, can facilitate As methylation and elimination.

## 1. Introduction

Arsenic (As) is a naturally occurring element commonly present in environmental sources such as air, water, and soil [1]. Through processes that are incompletely understood, As in soil can be mobilized leading to enrichment of As in groundwater. While drinking water is the most common source of exposure, other sources include As from mining and smelting, wood preservatives, pesticides, and foods irrigated and/or prepared with As-contaminated water. Current estimates suggest that roughly 140 million people in Bangladesh, India, Vietnam, Nepal, and Cambodia are drinking water with As concentrations up to 100 times the World Health Organization (WHO) and USA Environmental Protection Agency (EPA) guideline of 10 *μ*g/L [2, 3]. Chile, Mexico, China, and Taiwan also have As in groundwater that is used for drinking. In comparison to the situation in South and East Asia, the magnitude of the problem in the USA is relatively small. Nevertheless, the US Geological Survey estimates that 42 million Americans obtain their drinking water from household wells, and roughly 15% of these wells exceed the WHO guideline, indicating that a large number of USA residents are exposed to excess As from household wells [4]. In addition, not all municipalities are yet in compliance with the EPA requirements, with up to 8% of all public water supplies exceeding 10 *μ*g As/L.

Individuals chronically exposed to As are at increased risk for various cancers, including cancers of the skin (Bowen's disease, basal cell carcinomas, and squamous cell carcinomas) [5], lung, bladder, and liver [6]. Chronic As exposure is a major risk factor for ischemic heart disease [7] and Blackfoot Disease, the latter a form of severe peripheral vascular disease associated with systemic atherosclerosis, dry gangrene, and spontaneous amputations of affected extremities [8].

Arsenic is metabolized via methylation and understanding of the importance of As methylation has advanced substantially in the past decade. This paper provides an overview of what is now known about As methylation, including the details of the methylation pathway and the influence of methylation on As elimination, toxicity, and risk for As-induced health outcomes. We will also discuss the accumulating evidence suggesting that nutritional manipulation of one-carbon metabolism can improve As methylation capacity.

### 1.1. As Methylation

**Figure 1:**
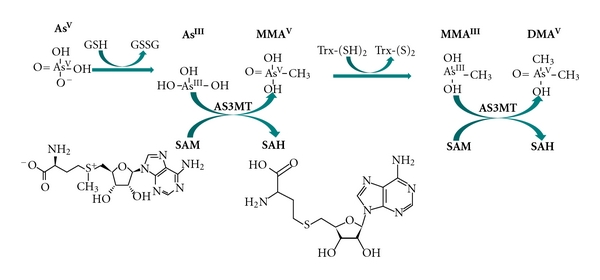
Arsenic metabolism. Arsenic in tubewells in Bangladesh is predominantly arsenite. Arsenic (+3 oxidation state) methyltransferase (AS3MT) catalyzes the oxidative methylation of arsenite using s-adenosylmethionine (SAM) as the methyl donor, forming methylarsonic acid (MMA^V^), and s-adenosylhomocysteine (SAH). MMA^V^ is reduced to methylarsonous acid (MMA^III^) before a subsequent oxidative methylation step yielding dimethylarsinic acid (DMA^V^) and SAH.

The predominant forms of As in drinking water are arsenate (As^V^) and arsenite (As^III^). Most species methylate these inorganic As (InAs) species to varying degrees in a process commonly thought to involve alternate reduction and oxidative methylation reactions in a model originally proposed by Challenger roughly 70 years ago [9, 10] (Figure 1). In this model, InAs^III^ serves as a substrate for As methyltransferase (AS3MT), an enzyme identified by Thomas's group in 2002 [11, 12]. AS3MT catalyzes the oxidative methylation of InAs^III^ to methylarsonic acid (MMA^V^) using S-adenosylmethionine (SAM) as a cosubstrate. MMA^V^ is reduced to MMA^III^ in a reaction that can be catalyzed by AS3MT using reducing equivalents provided by thioredoxin [13]. Although earlier reports identified MMA^V^ reductase, a member of the glutathione-S-transferase superfamily (GSTΩ) [14, 15] as being capable of catalyzing this reduction, studies employing GSTΩ knockout mice indicate this function is not unique to GSTΩ (Chowdhury UK 2006). The methylation of MMA^III^ by AS3MT yields dimethylarsinic acid (DMA^V^), which is considerably less toxic than pentavalent or trivalent InAs or MMA. While other methyltransferase enzymes have been identified which are capable of catalyzing these methylation reactions [16, 17], *AS3MT* catalyzes the formation of MMA^V^ and DMA^V^ with a kM in the nM range [12]. Although methylation of As is generally considered to be primarily hepatic, AS3MT mRNA has also been detected in rat kidney, adrenal gland, lung, urinary bladder, heart, and brain [12]. 

There are controversies related to As metabolism and toxicity that warrant discussion. For example, DMA^V^ can be reduced, and some investigators have reported that DMA^III^ may represent a significant proportion of total urinary As [18–20]; however the potential for artifact is high for several reasons [21]. First, DMA^III^ in urine is highly labile; Gong et al. have demonstrated that it is completely oxidized to DMA^V^ within 24 hours when stored at −20°C [22]. Second, the method employed in these studies [18, 19] to synthesize the purported chromatographic standard for DMA^III^, that is, treatment of DMA^V^ with metabisulfite and thiosulfate, in fact does not generate DMA^III^, but rather thio-DMA^V^ [23]. Third, while Valenzuela et al. used appropriate DMA^III^ standards [20], the hydride analyte ultimately detected by their assay could be generated from either DMA^III^ or thio-DMA [21]. This thioarsenical species has recently been found to be present as a relatively minor (roughly 5% of total urinary As) arsenical species in urine of Bangladeshi women [21]. For these reasons, it is likely that the chromatographic peak assigned as DMA^III^ in population studies was actually thio-DMA^V^. While the toxicity of thio-DMA^V^ has not been well characterized, Francesconi's group has shown that thio-DMA^V^ reduces HepG2 cell viability to a greater extent than DMA^V^ at concentrations ranging from 0.1 to 1 mM [21]. A lesser issue relates to trimethylarsine oxide which can be formed under some assay conditions, but its production is strongly inhibited by the presence of GSH [11], and measurable amounts are generally not thought to be produced by humans *in vivo*.

A second model of As methylation proposed by Hayakawa et al. in 2005 suggests that trivalent arsenical-thiol complexes are the obligate substrates for AS3MT which catalyzes nonoxidative methylation reactions [24]. Work by Naranmandura and colleagues suggests a reductive methylation brought about by the transfer of an unshared electron pair from As to a methylation and concurrent reduction of As by conjugated GSH [25]. Future studies characterizing the structure and function of the AS3MT enzyme will be required to fully characterize the details of this metabolic process.

### 1.2. The Role of Methylation in As Elimination

Recent work by Thomas's group using AS3MT knockout mice has confirmed the crucial role of AS3MT in the methylation and excretion of As. After a single oral dose of 0.5 mg of arsenic as arsenate per kg body weight, AS3MT knockout mice showed a reduced proportion of methylated arsenicals in both liver and urine at 2 and 24 hours compared to wild-type mice [26]. AS3MT knockout mice also retained a higher percentage of the initial body burden of As in the liver, kidneys, urinary bladder, lungs, heart, and carcass at 24 hours than did wild-type mice. In a subsequent study in which mice received 10 daily oral doses of 0.5 mg of arsenic as arsenate per kilogram body weight, the AS3MT knockout mice showed reduced whole body clearance of As 24 hours after the final dose compared to wild-type mice (40% versus 90% clearance) [27]. During the clearance phase, DMA accounted for 6.9% of urinary As at 240 hours after the first dose in the knockout mice as compared to 84% in the wild-type mice. The AS3MT knockout mice also showed higher fractions of the body burden of As in skin, liver, and urinary bladder as compared to wild type. Although the AS3MT gene resulted in a substantially reduced capacity for As methylation, methylated arsenicals were still observed in urine and tissues of AS3MT knockout mice. In fact, the AS3MT knockout mice had a higher proportion of MMA in both urine and liver than did wild type [26]. In a study which underscores the critical role of AS3MT in As excretion, knockout mice exposed to 100 or 150 ppm arsenite via diet showed severe, and often lethal, systemic toxicity after only one week of exposure [28]. Given that MMA^III^ is the most toxic As metabolite, an interesting question that arises from this work is whether the toxicity observed in the knockout mice is at least in part due to the increased proportion of MMA. The work of Thomas and colleagues also strongly suggests that there are alternate pathways for methylation of InAs to MMA. Indeed, this group recently reported the in-vitro conversion of arsenate to oxy- and thioarsenicals, including MMA^V^, by anaerobic microbiota of mouse cecum [29]. There may also be other enzymes capable of catalyzing these reactions.

The relative toxicities of the different As species are related to their chemical reactivity, but also to their physiologic half lives. Human retention studies employing single oral doses of ^74^As as arsenic acid or AsO(OH)_3_ to human volunteers indicate that arsenicals are eliminated with a three-component exponential decay pattern: 65.9% with a half life of 2 days, 30.4% with a half life of 9.5 days, and 3.7% with a half life of 38 days [30]. However, when considering populations that have been exposed to InAs for many years, these half lives derived from single-dose experiments must be viewed with caution since steady-state tissue concentrations were not achieved, and a “deep compartment” with a much longer half life could have been missed. Indeed, in mice, some InAs deposits in bone [31], suggesting that a longer terminal half life is likely. Similar patterns of elimination are observed in rabbits and hamsters. The initial half lives of MMA and DMA in hamsters are very short (7.4 and 5.6 h, respectively) [32], indicating the importance of As methylation for the facilitation of As elimination. The specific half lives of MMA and DMA in humans have not been determined, and their half lives under steady-state conditions may differ from those calculated using tracer kinetics with single oral doses. Somewhat remarkably, our understanding of the renal mechanisms of As excretion is limited to early studies relating transport of arsenate (InAs^V^) to phosphate in dogs [33–35]; little is known about renal excretion and/or potential reuptake of different As metabolites.

### 1.3. Mechanisms of Action of As

InAs is a highly reactive metalloid. As^III^ toxicity is largely attributable to its ability to react with critical sulfhydryl groups of many enzymes. The complex of As with a given protein bestows selectivity to the biological effects of As [36] and As metabolites differ in their protein binding capacity: InAs^III^ has three coordination sites, MMA^III^ has two, and DMA^III^ has only one [37]. A stable structure only forms when As complexes with two sulfhydryl groups in a single protein. For this reason, the stability and specificity of binding between DMA and monothiols are less than that formed between InAs^III^ or MMA^III^ and dithiols [36]. Toxicities of As^V^ are related to its striking chemical resemblance to phosphate. For example, As^V^ can serve as a substrate for enzymes that normally utilize phosphate, potentially resulting in disruption of normal biochemical processes [38].

Although As is an established human carcinogen, the carcinogenic mechanism of As is likely to be through a novel process, as As is a poor mutagen in *in vitro* studies. As a notable exception, Waalkes group has determined that *in utero* As exposure during critical periods of development results in various tumors in the adult offspring, indicating that in these circumstances As can act as a complete carcinogen. The carcinogenic mechanism of action of As is not known, but may involve oxidative stress and clastogenicity [39, 40]. Arsenic is considered to be a member of a class of carcinogens known as gene inducers, or indirect carcinogens, due, in part, to its proposed influence on DNA methylation [41]. Recent evidence also indicates that As exposure may be associated with alterations in histone modifications [42–58] and that *in utero* exposure is associated with alterations in stem cell response to carcinogen exposure during adulthood [59].

### 1.4. Arsenic Methylation as a Detoxification Pathway

Despite decades of research implicating As methylation as a detoxification pathway, the influence of As methylation on As toxicity has been under intense investigation in recent years. Landmark work by Styblo et al. [60] and Petrick et al. [61, 62] in 2000 found MMA^III^ to be the most toxic metabolite both *in vitro* and *in vivo.* Subsequent toxicological studies have confirmed that MMA^III^ and DMA^III^ are at least as cytotoxic [63] and genotoxic [64–68] as InAs^III^. In contrast, data suggesting that DMA^V^ is a bladder carcinogen in rats [69] has been discounted in terms of human relevance due to the extraordinarily high doses employed [70]. Also, animal models suffer limitations in that there are profound species differences in As metabolism, and animals are less prone to develop cancer in response to As exposure than humans. Based largely on cell culture studies, the relative toxicities are thought to be MMA^III^ > DMA^III^ > InAs^III^ > InAs^V^ > MMA^V^ > DMA^V^, with the qualification that DMA^III^ is highly unstable and is not likely present in significant quantities *in vivo* [21].

The relatively high toxicity of MMA^III^ leads to a critical question as to whether the overall methylation process is one of detoxification or of bioactivation since this metabolite is thought to be a requisite intermediate in the generation of DMA^V^. Under conditions of chronic low-dose InAs exposure, the extent to which MMA^III^ arising from endogenous biosynthesis exists as a free moiety within the intracellular milieu as opposed to that which is bound to GSH, AS3MT, or other cellular proteins is an open question and would likely influence its toxicity. Indirect evidence of a beneficial effect of methylation comes from studies indicating a protective role for folate and/or SAM or of enhanced As toxicity under conditions of folate deficiency. For example, McDorman et al. demonstrated that dietary folate deficiency enhances the induction of As-induced micronuclei by 1.3- to 4.5-fold as compared to folate sufficient mice at As doses of 2.4 to 10 mg/kg via oral gavage [71]. Likewise, Ramirez et al. [72] induced micronuclei formation in human lymphocytes with 10 *μ*M sodium arsenite and found that micronuclei formation was attenuated by the addition of 17 nM SAM. Folate deficiency has also been reported to enhance the effects of As on gene expression in a study employing skin biopsies from K6/ODC mice, one of the few rodent models sensitive to As-induced tumorigenesis [73]. While suggestive, these studies lack direct measures of As metabolites, and alternative explanations for the observations cannot be ruled out.

In human populations, case-control studies indicate that individuals with relatively higher proportions of MMA^(III+V)^ and lower proportions of DMA in urine are at increased risk for As-related health outcomes, including skin lesions, skin, lung, and bladder cancers, peripheral vascular disease, and atherosclerosis [5, 74–85]. Note that technology does not yet allow for reliable speciation of MMA^III^ versus MMA^V^ as MMA^III^ is very readily oxidized to MMA^V^ during sample collection, storage, and processing. Some of the above studies were limited in that the number of cases was relatively small (i.e., 26 to 76 cases) [75, 76, 78, 79] and while the odds ratios were not inclusive of one, the 95% confidence intervals tended to be relatively wide (e.g., 1.7–34 for skin cancers) [75]. Two larger studies include one case-control study of urothelial carcinoma (*N* = 177 cases versus 488 controls) in Taiwan and another study of skin lesions (*N* = 594 cases versus 1,041 controls) in Bangladesh. The Taiwan study found that total urinary As, %InAs and %MMA all exhibited significant dose-dependent increased risk for urothelial carcinoma, whereas %DMA was associated with decreased risk [77]. The Bangladesh study found that %MMA in urine was positively associated with risk for skin lesions in a dose-dependent manner, while %DMA was inversely associated with risk [74]. Thus, the weight of the human evidence favors the consensus that incomplete methylation of As to DMA confers increased susceptibility to multiple adverse health outcomes. However, these studies all suffer the common limitation that As metabolites were assessed after disease onset, bringing into question the issue of temporality. In sum, although there appears to be an emerging consensus among epidemiologists that complete methylation of As to DMA is beneficial, the lack of large scale population-based studies analyzing prediagnostic biological samples renders this consensus somewhat tenuous.

## 2. Nutritional Influences on Methylation Reactions

**Figure 2:**
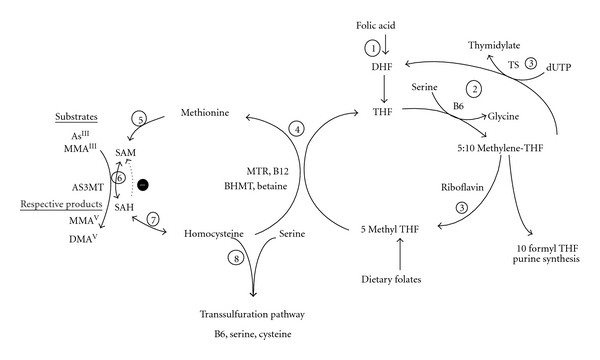
One-carbon metabolism. (1) Folic acid, arising from fortified foods or nutritional supplements, is reduced to dihydrofolate (DHF) and tetrahydrofolate (THF) by dihydrofolate reductase. (2) Serine hydroxymethyl-transferase transfers 1-carbon units from serine to THF, with PLP as a coenzyme, forming 5,10-methylene-THF and glycine. (3) 5,10-methyl THF reductase can reduce 5,10-methylene-THF to 5-methyl-THF. 5,10-methylene-THF can also generate DHF during the synthesis of thymidylate. After absorption from the GI tract, dietary folates can also enter the one-carbon metabolic pathway as 5 methyl THF. (4) In a reaction catalyzed by methionine synthetase and utilizing vitamin B12 as a cofactor, the methyl group of 5-methyl-THF is transferred to homocysteine (Hcys), generating methionine and THF. Alternatively, betaine can donate a methyl group for the remethylation of homocysteine to methionine in a reaction catalyzed by betaine homocysteine methyltransferase (BHMT). (5) Methionine adenosyltransferase activates methionine to form S-adenosylmethionine (SAM). (6) SAM is a methyl donor for a variety of acceptors, including guanidinoacetate (GAA—precursor to creatine), DNA, and As, in reactions that involve a number of methyltransferases. (7) The byproduct of these methylation reactions, s-adenosylhomocysteine (SAH), is hydrolyzed to generate Hcys. (8) Hcys is either used to regenerate methionine or is directed to the transsulfuration pathway.

Methylation of As and numerous other substrates occurs via one-carbon metabolism, a biochemical pathway important in the biosynthesis of purines and thymidylate and the remethylation of homocysteine (Hcys) to methionine (Figure 2). Methionine is activated to SAM, which serves as a methyl donor for a variety of methylation reactions, including the methylation of As. Transmethylation reactions generate s-adenosylhomocysteine (SAH), which can be converted to Hcys. SAH is a strong inhibitor of most methyltransferase enzymes, including AS3MT. One-carbon metabolism is dependent upon folate, vitamin B12, and vitamin B6 for the recruitment and transfer of methyl groups. Other nutrients, including betaine, choline, riboflavin, and serine also contribute to the availability of methyl groups ultimately used in SAM biosynthesis.

### 2.1. Experimental Evidence of Nutritional Influences on As Methylation and Toxicity

Early studies observed effects of methyl donor deficiency on As excretion and provide experimental evidence that the well-characterized nutritional regulation of one-carbon metabolism can influence As methylation and toxicity. In 1987, Vahter and Marafante reported that methyl donor deficiency in rabbits induced by either choline-, methionine- or protein-deficient diets significantly decreased urinary excretion of As, mainly due to lower DMA excretion. These diets also gave rise to increased retention of As in tissues (e.g., lung) [86], suggesting longer half lives and greater chemical reactivity of the InAs species. Similarly, Tice et al. reported that methyl donor deficiency induced by a choline-deficient diet decreased total urinary As excretion in mice by 28% as compared to mice on a choline-sufficient diet, predominantly due to reduced urinary DMA [87]. This was also accompanied by a shift in target organ As-induced DNA damage from liver and bladder (sites of As methylation and urinary As elimination, respectively) to skin [87], a target tissue in which As has a high affinity for the sulfhydryl groups of keratin.

An elegant series of studies by Finnell's group on As-induced NTDs employed mice heterozygous or nullizygous for folate binding proteins including Folbp-1, -2, and reduced folate carrier (RFC) [88–91]. Each of these binding proteins functions in cellular uptake of folate from the circulation (Folbp1 and 2) and/or enterocytes (RFC). While mice nullizygous for Folbp1 and RFC die *in utero*, the heterozygotes and Folbp2−/− develop normally and Folbp1 embryos can be rescued with folinic acid. The study protocols employed i.p. injections of sodium arsenate (30–40 mg/kg) on gestational days 7.5 and 8.5, that is, a critical period for neural tube closure. These studies demonstrated that for all genotypes studied (including wildtype), dietary folate deficiency caused a reduction in total urinary As excretion, primarily due to a reduction in DMA excretion. Furthermore, folate-binding protein 2−/− mice were more susceptible to As-induced NTDs, a phenotype that was further exacerbated by a folate-deficient diet [90].

While these studies provide strong experimental evidence that nutritional manipulation of one-carbon metabolism influences As methylation, excretion, and toxicity, the As doses were high, and the dietary deficiencies were severe. Moreover, there are marked species variations in the efficiency of As methylation [92].

### 2.2. Nutritional Influences on As Methylation in Humans

As noted, rodent models suffer two limitations: there are profound species differences in As metabolism, both mice and rats are extremely efficient in methylation of As, and animals are less prone to develop cancer in response to As exposure than humans. Furthermore, it is difficult to mimic chronic low-dose population exposure levels using rodent models. The earliest human data implicating nutritional influences on methylation and toxicity of As came in the form of isolated case reports. For example, there is an interesting case study of a girl with MTHFR deficiency who developed severe clinical signs and symptoms of As poisoning upon exposure to an As-containing pesticide, whereas no other exposed family members developed symptoms [93]. In 2002, in a study of 11 families in Chile, Smith's group reported intra-family associations in As methylation. The father/mother correlation for In As/(MMA+DMA) was low (*r* = 0.18). However, adjustment for plasma folate or homocysteine substantially increased the correlations (*r* = 0.33 and 0.55, resp.) [94]. Although the authors did not conclude that there was a significant effect of nutritional factors on As methylation, the data were highly suggestive.

In 2005, this same group assessed dietary intake of 30 micronutrients by dietary questionnaire in a sample of 87 subjects from two As-exposed regions in the western USA. They found that subjects in the lower quartile for dietary protein, iron, zinc and niacin had higher %MMA and lower %DMA than subjects in the higher quartile [95]. No associations were found for dietary folate, but the study was conducted several years after mandatory folic acid fortification of the USA food supply and therefore all of the study subjects were essentially folate supplemented.

More recently, Vahter's group analyzed plasma concentrations of folate, cobalamin, zinc, and ferritin in a cross-sectional study of 442 pregnant women from Matlab, Bangladesh. In their analyses, they first stratified by As exposure (tertiles of urinary As) and then compared %InAs, %MMA, and %DMA across tertiles of plasma micronutrient concentrations. In a multivariate adjusted model, only %InAs was found to be lower with increasing plasma folate concentrations and only among the highest As exposure subgroup [96]. In a subsequent study in a subset of 324 women from the same parent study who had urine samples available at gestational weeks 8, 14, and 30, Vahter et al. examined changes in As methylation during the course of pregnancy and whether any observed changes were associated with nutritional status. Gestational week was inversely associated with the percentage of urinary InAs and positively associated with the percentage of urinary MMA and DMA (*P* < 0.001 for all). There were no observed associations between plasma folate or vitamin B12 and the change in urinary %InAs, %MMA, and %DMA over the course of pregnancy [97]. Although the authors concluded from these studies that nutritional status had little influence on As methylation, this is not surprising given that plasma folate concentrations change dramatically over the course of pregnancy, introducing noise to the variable. Also, all of these women had been given prenatal folic acid (400 *μ*g) starting at week 14 in addition to other vitamin and/or mineral supplements. In addition, it is possible that other nutrients which were not examined in this study, such as choline and betaine, play a more substantial role in As methylation during pregnancy; choline biosynthesis is significantly upregulated by estrogen, concentrations of which rise dramatically during pregnancy [98].

Our group has conducted a series of studies in Bangladesh on nutritional influences on As metabolism and toxicity. We first evaluated the underlying prevalence of folate and B12-deficiency and hyperhomocysteinemia (HHcys) in a random sample of 1,650 Bangladeshi adults. This survey revealed that the study population has an extremely high prevalence of HHcys, particularly among males: 63% of males and 26% of females were found to have hyperhomocysteinemia (using NHANES cutoffs of ≥11.4 and 10.4 *μ*mol/L, for males and females, respectively) [99]. The data are consistent with a 2000 report in Lancet. In that study of healthy males, plasma total homocysteine (tHcys) concentrations were higher in Indian Asian men residing in the UK than their white European counterparts [100]. Our survey also revealed modest but statistically significant negative correlations between water As and plasma folate concentrations (*r* = −0.13, *P* > 0.0001), suggesting that As may in some way negatively impact folate nutritional status.

We subsequently selected a subset of 300 participants from the survey for measurement of urinary As metabolites for a cross-sectional study on the associations between folate, tHcys, and As methylation [101]. This subset was selected to be representative of the study population for total urinary As after excluding those identified as being cobalamin deficient. The results of these analyses revealed moderate but significant positive correlations between plasma folate and the relative proportion of DMA (%DMA) in urine and negative correlations between folate and both InAs and MMA in urine (Spearman Correlations −0.12, −0.12, and 0.14, for %InAs, %MMA and %DMA, resp.; *P* < 0.05 for all). Concentrations of tHcys were positively correlated with %MMA (*r* = 0.21, *P* < 0.001) and negatively correlated with %DMA (*r* = −0.14, *P* < 0.001).

In this same study, we made the serendipitous observation that urinary creatinine is negatively correlated with %InAs and positively correlated with %DMA in urine (*r* = −0.32 and 0.30, resp., *P* > 0.0001); the correlations remain equally robust with and without control for covariates including body weight, age, and water or urine As concentrations. We have confirmed this observation in several subsequent studies in Bangladesh [53, 102–104] and in an unpublished analysis of data from adults in Mexico provided from Drs. Uttam Chowdhury and H. Vasken Aposhian. Smith's group has subsequently reported similar findings in West Bengal [105]. The underlying mechanism for this observation is not readily apparent, but we note the substantial role of creatine biosynthesis on consumption of SAM-derived methyl groups [106], a role that is downregulated with increased dietary creatine intake.

In 2006, we reported the initial findings from our randomized, controlled trial of folic acid supplementation [103]. For this trial, 200 participants were randomly selected from the 550 participants who fell into the lowest tertile for plasma folate in the survey of 1,650. Participants were excluded if they were cobalamin deficient, pregnant, or taking vitamin supplements. Participants were randomly assigned to receive folic acid (400 *μ*g/day, that is, the USA RDA) or placebo for 12 weeks. Urinary As metabolites were measured at enrollment, after one week and after 12 weeks. Folic acid supplementation resulted in an increase in the proportion of total urinary As excreted as DMA (72% before and 79% after) that was significantly (*P* < 0.0001) greater than that in the placebo group, as was the reduction in %MMA (13% before and 10% after, *P* < 0.0001) and %InAs (15% before and 11% after, *P* < 0.001). Significant treatment group differences were also observed for %MMA and %DMA even after just one week of the intervention.

Based on our understanding that As methylation facilitates urinary As elimination, and our observation that folic acid supplementation increased As methylation, we hypothesized that increased As methylation with folic acid supplementation would lower blood As concentrations. Methodologic advances in our Trace Metals Core Laboratory permitted us to test this hypothesis using blood samples from our folic acid trial by measuring total As and As metabolites in blood, where concentrations are an order of magnitude lower (range: 3–29 *μ*g/L) than those in urine (8–780 *μ*g/L). We measured As metabolites in blood for 130 participants, that is, those participants from our previous trial who had detectable levels of all As metabolites. Results revealed that folic acid supplementation resulted in a decline in total blood As of 13.6 ± 2.9% as compared to 2.5 ± 3.2% for the placebo group (*P* = 0.01) [107]. The decline in blood As was largely due to the decline in MMA in blood. Whereas total blood As (i.e., InAs+MMA+DMA) declined, on average, by 1.7 *μ*g/L, 1.1 *μ*g/L of this was MMA; MMA declined by 22% from baseline.

### 2.3. Nutritional Impact on Risk for As-Induced Health Outcomes

In 2004, Smith's group conducted a dietary recall study in West Bengal, India, of 192 skin lesion cases and 192 age- and sex-matched controls. The results of this study indicated that participants falling into the lowest quintile for animal protein, calcium, fiber, and folate were at increased risk for As-induced skin lesions [108]. In 2006, this group reported results of a study of plasma concentrations of a series of 17 metabolites (including a series of micronutrients and cholesterol, glucose, glutathione, homocysteine, and transthyretin) and risk for As-induced skin lesions; plasma analyses were done on a subset of 180 of the original 192 cases. No statistically significant odds ratios were observed for any of the parameters studied, including folate and homocysteine. However, approximately half of the samples were stored at 4°C overnight and not aliquoted and frozen until the day after collection [109]. Since folate is highly unstable and would likely be degraded under these conditions, and homocysteine continues to be released into plasma by red blood cells after sample collection, the plasma concentrations for these (and other) metabolites cannot be considered to be accurate due to the sample handling procedures. Additional limitations include lack of statistical control for differences in As exposure which differed by case-control status and lack of As metabolite data.

One of the strongest studies to date on the impact of nutritional status on risk for As-induced health outcomes is the recent aforementioned case-control study of 177 urothelial carcinoma cases and 488 controls in a population in Taiwan exposed to low concentrations of As in drinking water. This study found that higher %DMA in urine and higher plasma folate concentrations were associated with decreased risk. In a multivariate-adjusted model, the odds ratios (95% CI) for increasing quartiles of plasma folate concentrations were 1.0 (referent), 0.33 (0.20–0.54), 0.22 (0.13–0.38), and 0.09 (0.04–0.19), *P*_trend_ < 0.0001. Furthermore, a significant interaction was observed between urinary As profiles and plasma folate in affecting urothelial carcinoma risk [77].

We have conducted a nested-case control study of 274 skin-lesion cases individually matched to controls for gender and age (within 5 years) and frequency matched for water As (within 100 *μ*g/L). The results of this study indicate that folate deficiency and HHcys are both associated with increased risk for skin lesions [52], as is genomic hypomethylation of leukocyte DNA and low urinary creatinine [Odds ratios (95% confidence interval) were 1.8 (1.1–2.9) for plasma folate <9 nmol/L, 1.7 (1.1–2.6) for HHcys, 1.8 (1.2–2.8) for DNA methylation < median, and 0.7 (0.5–0.8) for a fold increase in urinary creatinine]. Clearly, clarification of the mechanisms underlying urinary As and creatinine interactions warrants further study.

## 3. Conclusions

The known health effects of long-term exposure to As include an array of health outcomes and increased risk for mortality. In countries with widespread contamination of drinking water, such as Bangladesh, the full impact of this exposure may not be apparent for years to come. Although there has been considerable debate as to whether As methylation is a bioactivation or detoxification pathway, the collective body of evidence from both laboratory and epidemiologic research has clarified several points. First, As methylation to DMA is primarily catalyzed by AS3MT and plays a key role in modulating As excretion and toxicity. Second, a lower capacity to methylate As to DMA, as evidenced by higher proportions of InAs and MMA in urine and blood, is associated with increased risks of several As-associated diseases. Lastly, the methylation and elimination of As are influenced by nutrients involved in one-carbon metabolism, especially folate. Further research is needed to determine whether other nutrients similarly influence As methylation and at what levels of intake the greatest benefit can be obtained. While clearly As-mitigation is of primary importance, nutritional manipulation of one-carbon metabolism may provide an additional approach for lessening the burden of disease resulting from long-term As exposure.
